# Global research trends and mechanistic pathways linking micro- and nano plastics exposure to metabolic health: a bibliometric and evidence-mapping analysis (2013–2025)

**DOI:** 10.3389/fnut.2026.1831474

**Published:** 2026-05-26

**Authors:** Goh Khang Wen, Santhra Segaran Balan, Noor Azimah Ahmad, Azrina Zainal Abidin, Fezah Othman, Ruth Naomi

**Affiliations:** 1 Faculty of Business and Communication, INTI International University, Nilai, Malaysia; 2 Department of Diagnostic & Allied Health Sciences, Faculty of Health and Life Sciences, Management and Science University, Shah Alam, Selangor, Malaysia; 3 Department of Human Anatomy, Faculty of Medicine and Health Sciences, Universiti Putra Malaysia, Sedang, Malaysia; 4 Department of Biomedical Sciences, Faculty of Medicine and Health Sciences, Universiti Putra Malaysia, Serdang, Malaysia; 5 Institute of Ocean and Earth Sciences (IOES), Canseleri Universiti Malaya, Kuala Lumpur, Wilayah Persekutuan Kuala Lumpur, Malaysia

**Keywords:** bibliometric analysis, lipid metabolism, metabolic health, microplastics, nano-plastics, oxidative stress

## Abstract

**Background:**

The widespread presence of micro- and nanoplastics (MNPs) in the food chain has raised concerns regarding their potential contribution to metabolic diseases.

**Objective:**

However, a comprehensive synthesis of the intellectual structure, thematic evolution, and biomarker evidence linking dietary MNP exposure to metabolic health outcomes remains limited.

**Methods:**

This study conducted a bibliometric analysis of publications indexed in Scopus and Web of Science Core Collection (WoS) between 2013 and 2025. Bibliometrix/Biblioshiny and VOSviewer were applied for performance analysis, science mapping (co-occurrence, co-citation, bibliographic coupling), thematic evolution, and biomarker-domain mining. A biomarker–mechanism evidence matrix was developed to integrate mechanistic insights.

**Results:**

1,613 entries were found in WoS out of the 1,725 papers in the Scopus dataset, indicating consistent publication trends across databases. After 2019, there was a significant increase in annual research production, which was indicative of the expanding interdisciplinary attention to metabolic concerns linked with MNP. The publication landscape is dominated by environmental toxicology journals, which are increasingly contributing worldwide and have a large geographic concentration in China. Four important research areas were found by keyword co-occurrence analysis: mammalian metabolic toxicity, gut microbiota dysbiosis, aquatic ecotoxicology, and developing human health risk assessment. Oxidative stress and exposure pathways were identified as major themes by thematic mapping, while gut microbiota–metabolic interactions represent quickly developing research avenues.

**Conclusion:**

Human epidemiological evidence remains limited compared to animal mechanistic studies, highlighting gaps in realistic exposure assessment and standardized cardiometabolic biomonitoring.

## 1 Introduction

Plastic debris is widely distributed throughout terrestrial, freshwater, and marine ecosystems as a result of the exponential increase in worldwide plastic production over the past few decades. Since the middle of the 20th century, plastic production has been projected to have reached billions of tons, with a significant amount of that plastic ending up in the environment as a result of poor waste management and environmental persistence (1). Microplastics (<5 mm) and nanoplastics (<1 μm) are produced by fragmentation and degradation processes and are becoming more frequently found in soil, water, air, and biota (2, 3). It is now acknowledged that these particles are new pollutants of international significance.

In addition to environmental contamination, there are other ways that micro- and nanoplastics (MNPs) have made their way into the human food chain. Research suggests that food-contact materials, bottled water, shellfish, salt, and honey are possible dietary exposure sources (4, 5). The possibility of systemic exposure is further supported by the discovery of plastic particles in human biological matrices, such as blood and lung tissue (6, 7). Early study mostly concentrated on ecotoxicological effects and environmental occurrence, but more and more emphasis is currently being paid to the possible health effects of long-term food exposure.

According to recent experimental research, exposure to MNPs associated with the disrupt host physiological functions, especially in the gastrointestinal system. For particles that are consumed orally, the gut serves as the main interface, where interactions with intestinal epithelial cells and the local microbiota may take place. According to new research, microplastics can change host metabolic profiles in animal models, cause dysbiosis of the gut microbiota, and weaken the intestinal barrier (8, 9). Oxidative stress, inflammatory signaling, and disturbances in the metabolism of fats and carbohydrates are often associated with these changes (10, 11). These results raise questions about a possible connection between dietary MNP exposure and metabolic diseases such as metabolic syndrome, obesity, insulin resistance, and non-alcoholic fatty liver disease (NAFLD). Hundreds of millions of people worldwide suffer from metabolic diseases like obesity, insulin resistance, and non-alcoholic fatty liver disease (NAFLD), which constitute an increasing global health burden. Cumulative metabolic dysregulation may result from even low-level long-term exposure to new environmental pollutants such micro- and nanoplastics.

One important mechanism linking environmental exposures to systemic metabolic disorders is the gut-liver axis. In MNP-related toxicity investigations, oxidative stress amplification, cytokine-mediated inflammation, and dysregulated lipid homeostasis are often documented mechanisms. However, compared to bigger microplastics, nanoplastics may display different biological behaviors, such as increased cellular absorption and translocation, because of their smaller size and higher surface reactivity. Heterogeneity in particle characterization, dosage measurements, and biomarker selection makes cross-study comparison and risk assessment more difficult, even with growing mechanistic insights.

Even while the body of research on MNPs has grown significantly in recent years, it is still dispersed over the fields of environmental science, toxicology, microbiology, and metabolic health. The intellectual structure, theme evolution, mechanistic focus, and translational gaps of studies relating food-chain MNP exposure to metabolic outcomes have not yet been thoroughly mapped by a thorough bibliometric synthesis. Determining knowledge clusters, new hotspots, and priority research areas requires an understanding of the evolution of this discipline, from environmental contamination studies to microbiome-mediated metabolic frameworks.

Few research have explicitly looked at the relationship between dietary exposure to micro- and nanoplastics and metabolic health consequences, despite the fact that numerous bibliometric studies have investigated microplastic contamination. Additionally, the reliability of trend identification may be limited by the fact that many prior bibliometric analyses rely on a single database. Cross-validation of publishing trends and theme structures is made possible by integrating many bibliographic databases, such as Web of Science (WoS) and Scopus, which enhances coverage. As a result, merging these datasets offers a more thorough picture of the intellectual environment and new lines of inquiry in this quickly developing multidisciplinary topic.

**Figure 1:**
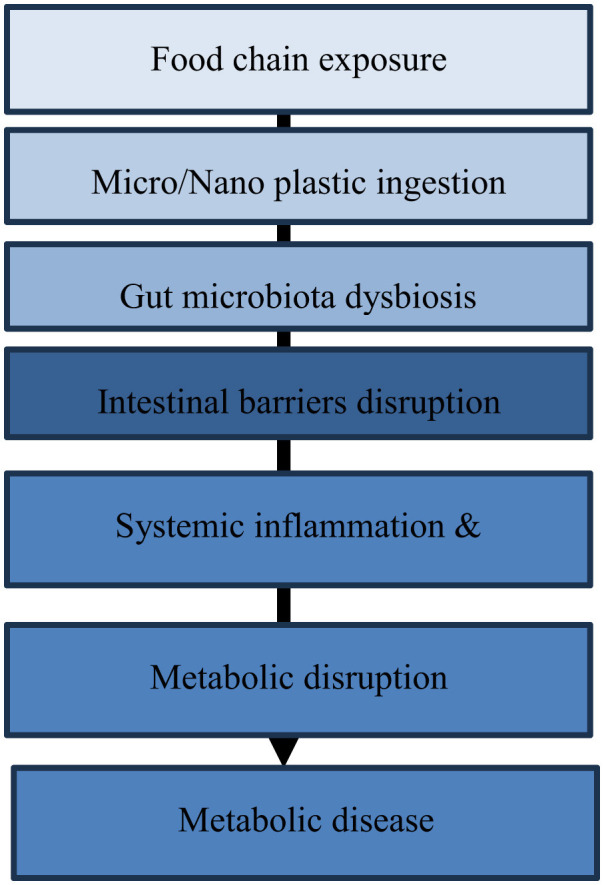
Conceptual framework illustrating proposed pathways linking dietary micro- and nanoplastic exposure to metabolic health disruption through gut microbiota dysbiosis, oxidative stress, inflammatory signaling, and metabolic pathway perturbation.

Thus, the goal of the current study is to provide a thorough bibliometric and mechanistic mapping of research on micro- and nanoplastics at the nexus of exposure to the food chain and metabolic health. In order to (i) characterize publication growth and global research distribution, (ii) identify major thematic clusters and emerging research fronts, (iii) investigate the field's intellectual underpinnings, and (iv) integrate biomarker-domain trends to highlight mechanistic convergence and translational gaps, we used performance analysis and science mapping techniques. This study offers a quantitative framework to guide future risk assessment, experimental standardization, and public health research objectives in the context of global plastic contamination by combining structural and mechanistic information (Figure 1). Figure 1 show the conceptual framework on proposed pathways linking dietary micro- and nanoplastic exposure to metabolic health disruption through gut microbiota dysbiosis, oxidative stress, inflammatory signaling, and metabolic pathway perturbation.

The current study focuses on combining bibliometric mapping with biomarker-domain evidence synthesis to link research trends with mechanistic pathways, in contrast to traditional bibliometric studies that mainly describe publication patterns only. The identification of translational gaps between experimental results and practical consequences for metabolic health is made possible by this dual analytical framework.

## 2 Materials and methods

### 2.1 Data collection

The Scopus and Web of Science Core Collection (WoS) databases were used to perform a comprehensive bibliometric analysis due to their broad coverage of peer-reviewed scientific literature across environmental, biomedical, and toxicological disciplines. Scopus provides broader coverage of interdisciplinary journals, while Web of Science offers high selectivity and citation indexing reliability. Combining both databases enhances dataset completeness and minimizes database-specific bias. For this study, the search was carried out in February 2026 to guarantee nearly full indexing of 2025 entries and comprised publications from January 2013 to December 2025.

The following search string was applied in both Scopus and Web of Science databases: TITLE-ABS-KEY((microplastic^*^ OR nanoplastic^*^ OR “micro plastic^*^” OR “nano plastic^*^” OR MNPL^*^ OR MNPs) AND (ingest^*^ OR dietary OR diet^*^ OR oral OR gastrointestinal OR intestinal OR gut OR “food chain” OR seafood OR fish OR shellfish OR salt OR honey OR milk OR tea OR bottled OR packaging OR “food contact”) AND (obes^*^ OR “insulin resistance” OR diabet^*^ OR “metabolic syndrome” OR NAFLD OR NASH OR steatosis OR “fatty liver” OR dyslipid^*^ OR adipos^*^ OR “gut microbiota” OR microbiome OR “oxidative stress” OR ROS OR inflammation OR cytokine^*^ OR glucose OR lipid^*^ OR cholesterol OR triglycer^*^)) AND NOT TITLE-ABS-KEY(wastewater OR “water treatment” OR coagulation OR membrane OR filtration OR sludge OR soil OR sediment OR river OR estuary OR foliar OR duckweed OR tobacco OR “plant uptake” OR mulching OR adsorption OR sorption OR photocatalysis OR biodegradation). The same conceptual search string was applied across both databases; however, minor syntax adaptations were made to ensure compatibility. Specifically, the search was conducted using the TITLE-ABS-KEY field in Scopus, while the equivalent Topic field (TS=) was used in Web of Science, which covers title, abstract, and author keywords.

Only peer-reviewed journal articles published in English were included. Reviews, conference papers, editorials, and non-English publications were excluded to ensure dataset consistency. Data were retrieved on 21 January 2026, and manual screening was conducted to ensure dataset accuracy and relevance.

### 2.2 Screening and eligibility

**Figure 2:**
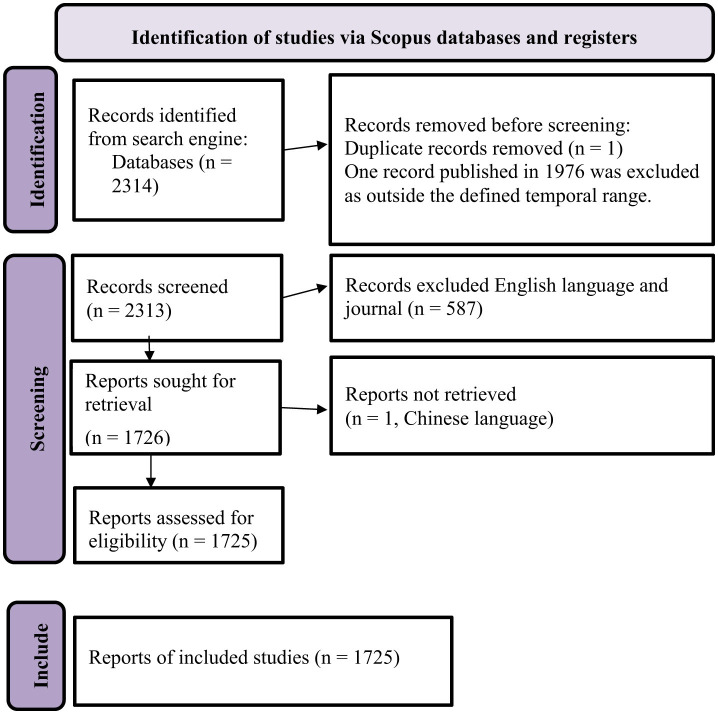
PRISMA flow diagram illustrating the identification, screening, eligibility, and inclusion process for the Scopus dataset used as the primary bibliometric source.

There were 2,314 records found in the first search. 588 records were eliminated following the application of language and document-type filters. One record that did not fall inside the specified time frame was eliminated. 1,725 publications were included in the final dataset for bibliometric analysis. A PRISMA-style flow diagram (Figure 2) illustrates the identification, screening, eligibility, and inclusion process. The PRISMA screening process was applied to the Scopus dataset, which served as the primary bibliometric source, while the Web of Science dataset was used for cross-database validation. The datasets were not merged. Duplicate records within each dataset were removed using Bibliometrix functions and manual verification. Screening was conducted independently by two reviewers using predefined inclusion criteria. Discrepancies were resolved through discussion until consensus was reached.

Inclusion criteria (i) Peer-reviewed journal articles, (ii) Published in English, (iii) Publication years between January 2013 and December 2025, (iv) Focus on micro- or nanoplastics in relation to ingestion, food-chain exposure, or gastrointestinal interaction and (v) Reporting metabolic, microbiome, oxidative, inflammatory, or lipid/glucose-related endpoints. Exclusion criteria (i) Conference papers, editorials, letters, book chapters, (ii) Non-English publications, (iii) Studies focused solely on environmental treatment technologies and (iv) Articles outside the defined temporal range.

The exclusion of environmental matrices (e.g., wastewater, soil, sediment) was intentional to restrict the analysis to studies focusing on dietary exposure pathways and metabolic health outcomes, thereby avoiding dilution of the dataset with environmental engineering and remediation studies. However, this may limit the representation of indirect exposure routes from environmental reservoirs to the food chain.

### 2.3 Data export and preparation

Complete records and cited references (authors, affiliations, titles, abstracts, keywords, source, year, and citation counts) were exported in CSV format. Before analysis, the consistency and completeness of the data were examined Duplicate records were identified and removed using automated filtering and manual verification to ensure dataset accuracy.

### 2.4 Bibliometric analysis

Bibliometric analyses were conducted using Bibliometrix/Biblioshiny (R package, version 4.5.2) for performance analysis and thematic mapping. Another app is VOSviewer (version 1.6.19) for network visualization and clustering. Two analytical approaches were employed:(i) Performance analysis is to evaluate the Annual scientific production, most relevant journals, country scientific production and institutional contributions. For Science mapping is to explore keyword co-occurrence networks, co-citation networks, bibliographic coupling and thematic map and thematic evolution. Network visualizations were generated using distance-based mapping algorithms in which node size represents frequency and link strength reflects co-occurrence or citation connectivity (12, 13). Keyword co-occurrence analysis applied a minimum threshold of 10 occurrences. Network normalization was performed using association strength, and clustering was conducted using default VOSviewer parameters.

### 2.5 Biomarker-domain mapping

Using a biomarker-domain mapping technique, mechanistic evidence was integrated. Using structured keyword mining of the dataset's titles, abstracts, and author keywords, biomarker domains (such as oxidative stress, lipid metabolism, glucose/insulin control, and inflammation) were found. This method does not amount to full-text biomarker extraction, but it does offer trend-level information. Biomarker-domain mapping was based on keyword mining of titles, abstracts, and author keywords, supplemented by manual curation.

### 2.6 Cross-database consistency analysis using Web of Science

A supplementary dataset was obtained from the Web of Science Core Collection (WoS) in order to improve the bibliometric analysis's robustness. WoS retrieval was conducted using the same search approach and inclusion criteria as the Scopus dataset. The Bibliometrix R software was used to integrate complete records and cited references that were exported in plain text format. 1,613 papers from the same time span (2013–2025) were included in the WoS dataset. To confirm publication trends, theme clusters, and intellectual structures, cross-database comparison was carried out. The trustworthiness of bibliometric results is strengthened by this multi-database validation method, which also lessens database-specific bias.

## 3 Results

### 3.1 Main information about the bibliometric dataset

**Figure 3:**
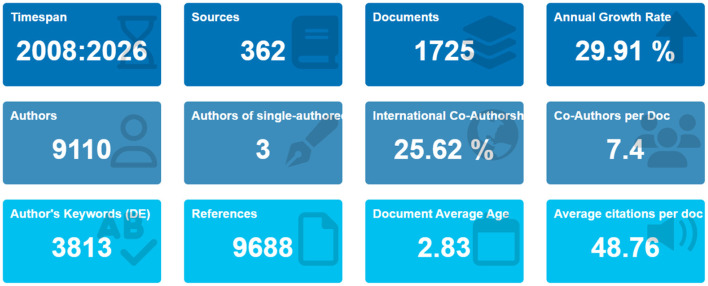
The main information of the refined documents.

Because of its wide coverage of peer-reviewed scientific literature, the Scopus database was used as the main dataset for the bibliometric analysis. The final dataset contained 1,725 peer-reviewed publications published between 2013 and 2025 after screening and eligibility evaluation. With an annual publication growth rate of 29.91%, these papers were dispersed among 362 scientific sources, demonstrating the field's rapid expansion. The dataset included 9,688 cited references, with an average of 48.76 citations per document, indicating the increasing scientific impact of research on the relationship between exposure to microplastics and metabolic health consequences (Figure 3). Figure 3 shows the main information of bibliometric dataset that we obtain for Scopus dataset.

### 3.2 Cross-database consistency analysis

**Figure 4:**
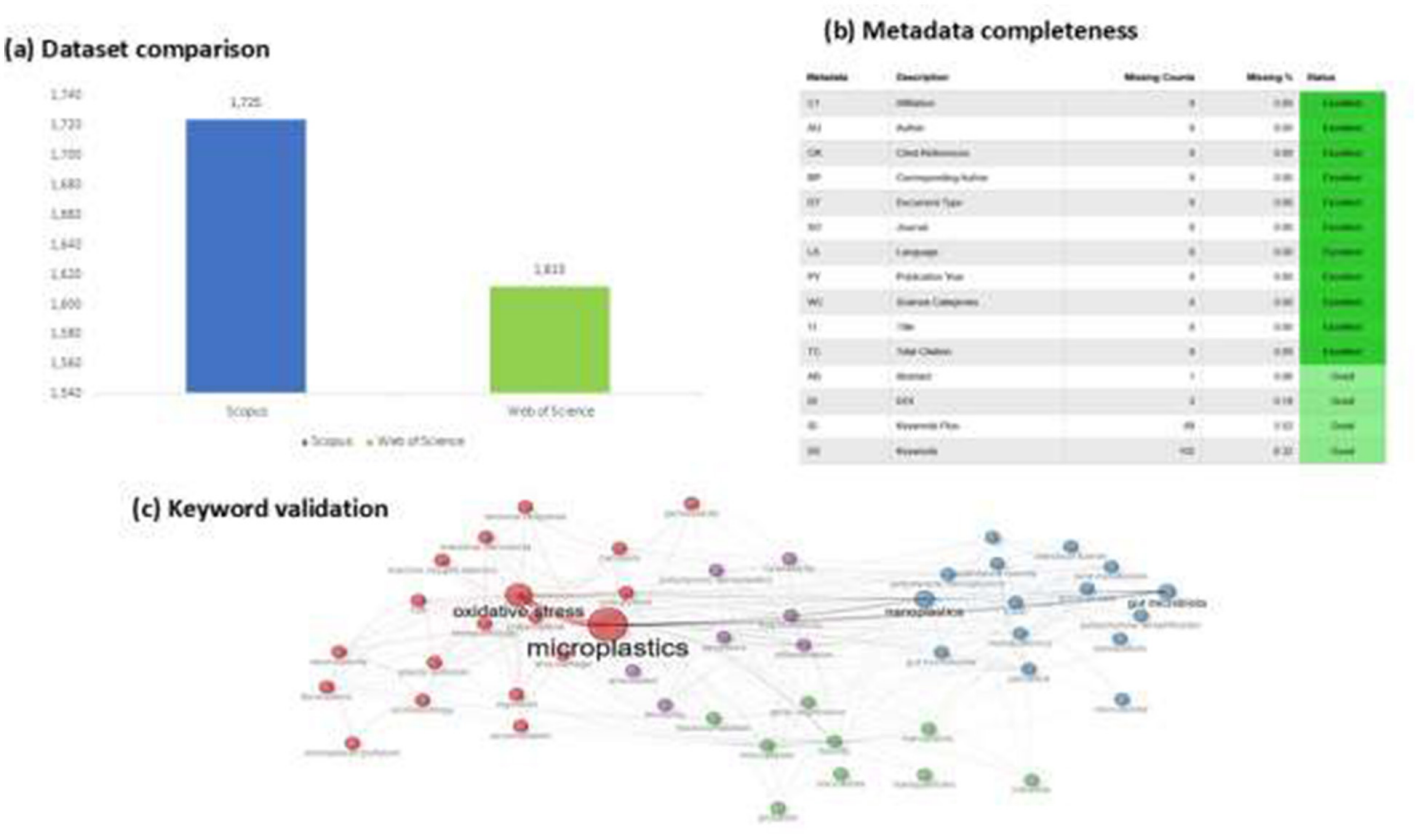
Cross-database consistency analysis of bibliometric datasets.

To validate the robustness of the bibliometric findings, a parallel dataset was retrieved from the Web of Science Core Collection. The 1,613 papers in the WoS dataset showed a high degree of agreement with the 1,725 entries in the Scopus dataset. High dataset dependability was indicated by metadata completeness analysis, which revealed few missing values in important categories like author information, cited references, and journal sources. Cross-database comparison demonstrated a high degree of consistency between Scopus and Web of Science datasets in terms of publication trends, thematic structures, and key research topics (Figure 4). Figure 4 shows the cross-data base consistency analysis from Web of Science (WOS).

### 3.3 Annual scientific production

**Figure 5:**
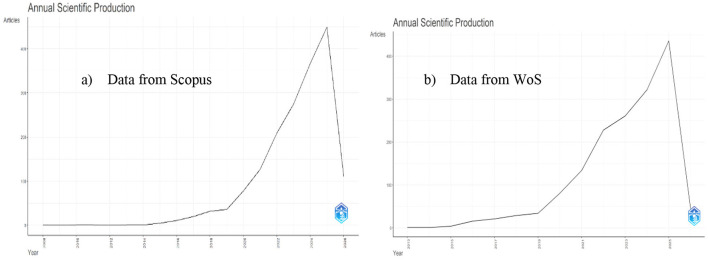
Annual scientific production of publications on micro- and nano-plastics in the food chain and metabolic health (2013–2025) from Scopus and WoS.

The yearly scientific output is displayed in Figure 5 (Scopus and WoS). According to the data, annual output stayed low for many years, but after 2019, it started to pick up speed, and in the most recent time, it increased until 2025. Due to incomplete year effects and indexing lag, it also displays the fall in 2026.

### 3.4 Most relevant journal

Environmental and toxicology journals accounted for the majority of publications, with Science of the Total Environment (*n* = 203) and the Journal of Hazardous Materials (*n* = 167) leading the pack, followed by Environmental Pollution (*n* = 132) and Ecotoxicology and Environmental Safety (*n* = 94). Increasing biological and health-oriented orientation is indicated by the existence of more mechanistic and biomedical channels (such as molecular and microbiological magazines) (Table 1).

**Table 1:** Most productive journals publishing research on micro- and nanoplastics and metabolic health.

Sources	Frequency
Science of the total Environment	203
Journal of Hazardous materials	167
Environmental Pollution	132
Ecotoxicology and Environmental Safety	94
Chemosphere	82
Aquatic Toxicology	65
Marine Pollution Bulletin	42
Environment International	41
Toxics	31
Environmental Science and Technology	28
International Journal of Molecular Sciences	25
Environmental Research	24
Comparative Biochemistry and Physiology Part - C: Toxicology and Pharmacology	21
Environmental Toxicology and Pharmacology	18
Environmental Science and Pollution Research	17

### 3.5 Country scientific production

**Figure 6:**
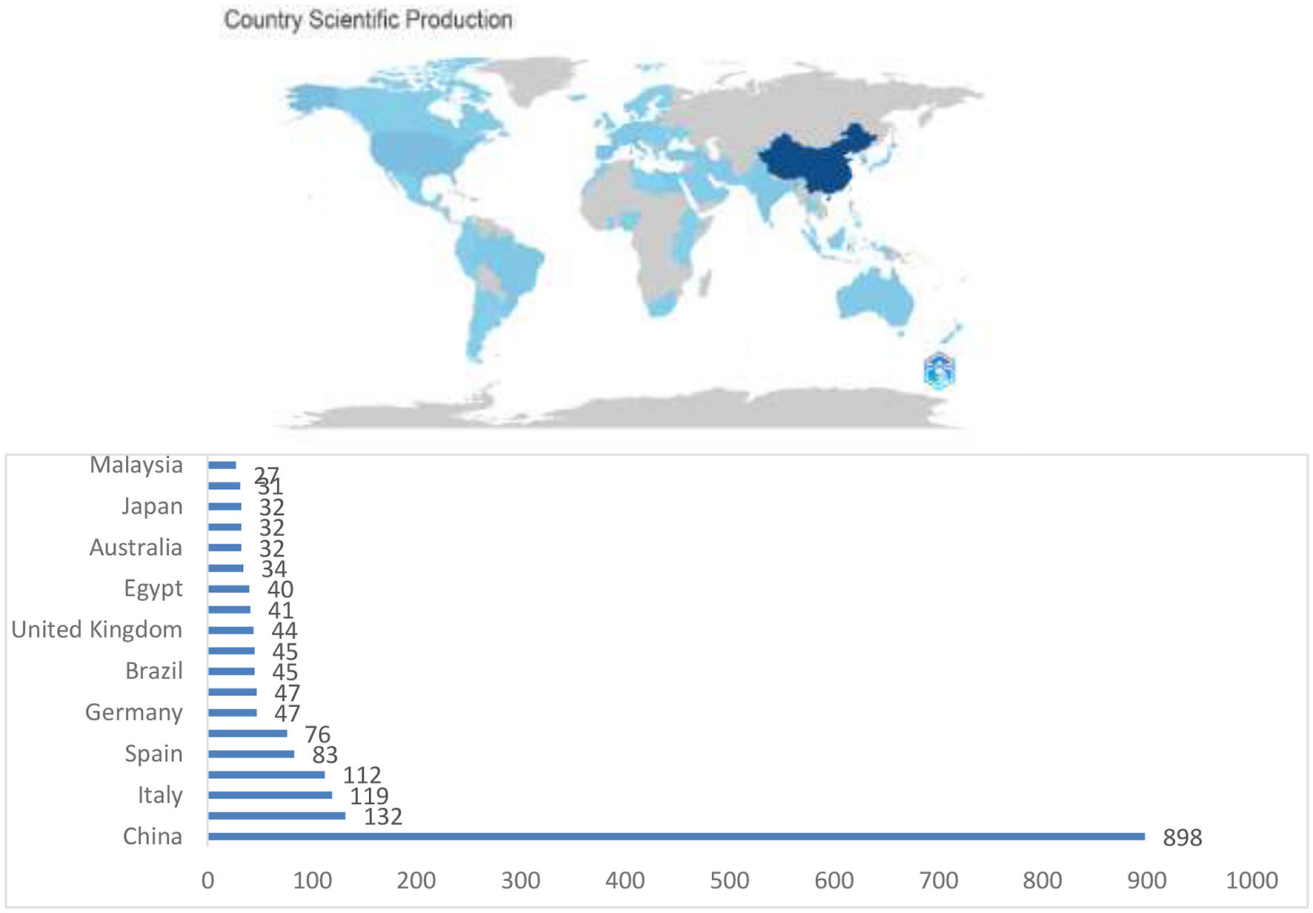
Global distribution of scientific production.

China (898) produced the most studies by a significant margin, demonstrating a clear dominance in the geographical distribution of publications. Next in line were South Korea (112), Italy (119), India (76), and the United States (132). Notable scientific activity was also shown by European nations such as Spain (83), Portugal (47), Germany (47), and the United Kingdom (44) as well. Malaysia shows emerging contribution but lacks translational metabolic studies (27), suggesting that regional interest in this field of study is developing. All things considered, the findings show robust Asian leadership with growing worldwide involvement (Figure 6). Figure 6 show the scientific production from Scopus dataset.

### 3.6 Affiliation analysis

China and other major research nations are the top affiliations publishing on micro-nanoplastics in food and metabolic health on human health, according to the top 10 institutional contributions analysis. South China Agricultural University (65), Southern Medical University (44), and Shanghai Ocean University (90) are the leading contributors in this discipline. This indicates that there is a lot of research being done in this field by Chinese institutions. Table 2 demonstrates that, despite the fact that other nations also contribute to this topic, the top 20 pertinent affiliations are found throughout Chinese universities.

**Table 2:** The most influential affiliation and its country.

Affiliations	Frequency
Shanghai Ocean University	90
South China Agricultural University	65
Southern Medical University	44
University Of Chinese Academy of Sciences	44
Nanjing University	42
Faculty Of Science	35
Ocean University of China	31
Universitat De Les Illes Balears	31
Capital Medical University	30
Chinese Academy of Sciences	29
Faculty Of Veterinary Medicine	28
Jinan University	27
Nanjing Normal University	26
Nanchang University	25
Universitat Autònoma De Barcelona	25

### 3.7 Co-authorship by country

**Figure 7:**
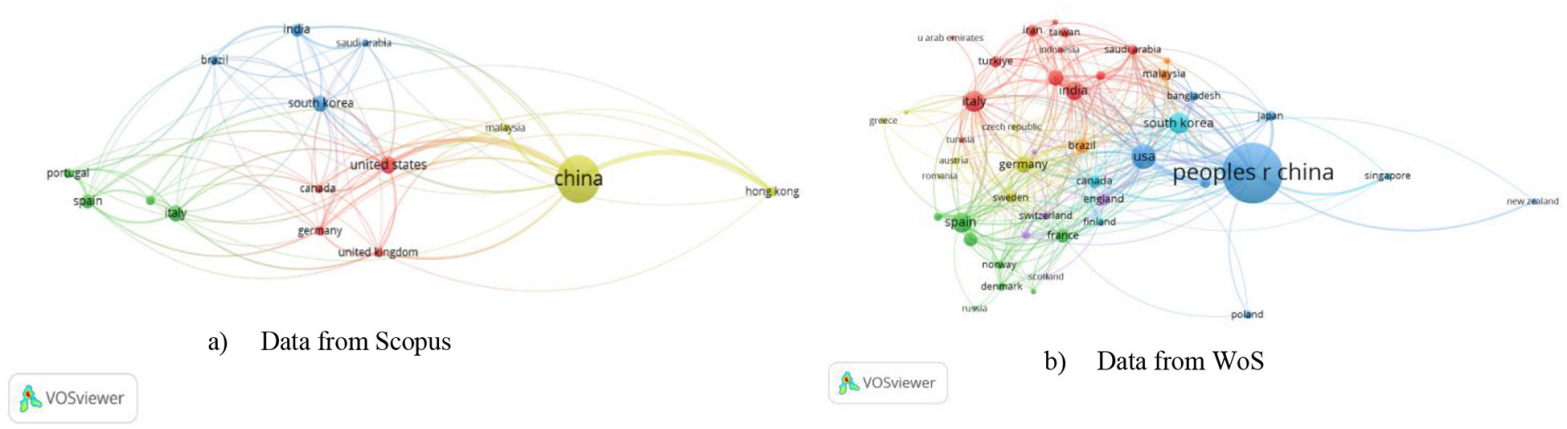
Global distribution of co authorship from Scopus and Wos.

Full counting was used for the co-authorship analysis by nation, and a minimum inclusion criterion was implemented. Figure 7 lists the top fifteen nations with the highest levels of co-authorship activity by overall link strength based on the data. With the highest total link strength (141), China was the most frequent provider, demonstrating its wide-ranging international cooperation. With total link strengths of 87 and 47, respectively, the United States and Spain ranked second and third. With link strengths of 34, 37, and 43, respectively, Italy, India, and South Korea were among the other top donors. The data show in the Web of Science (WoS) dataset, with China exhibiting the highest total link strength (195), followed by the United States (156) and Spain (88).

### 3.8 Thematic map analysis

**Figure 8:**
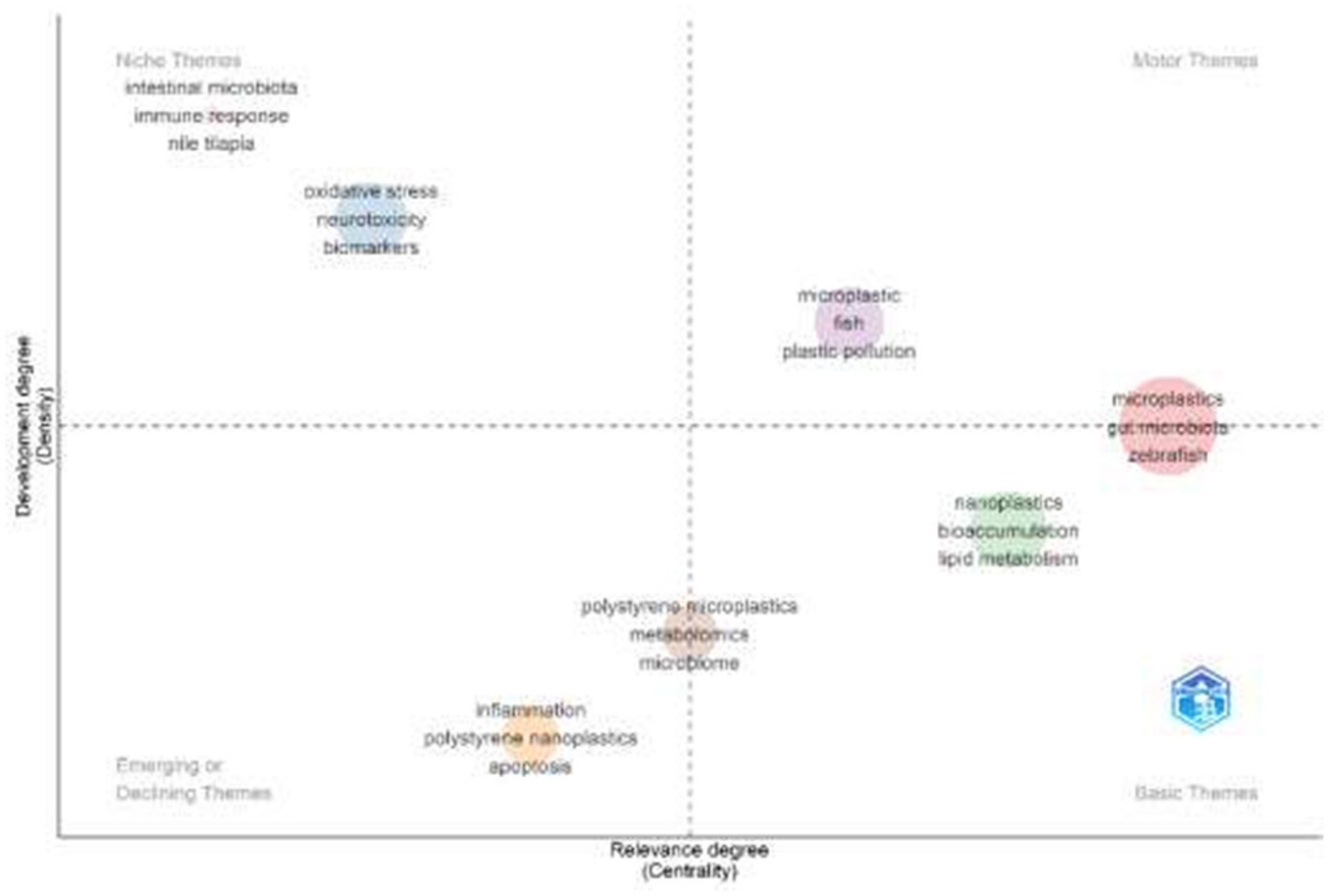
Thematic map based on author keywords showing conceptual structure of the field.

The gut microbiome–microplastics nexus is the main motor theme, according to the thematic map, suggesting a well-developed and important area of study. On the other hand, oxidative stress caused by nanoplastics and disturbance of lipid metabolism were shown to be highly pertinent but still unexplored fundamental issues. Conventional toxicological subjects like bisphenol A and environmental contamination developed become specialist areas with little connection to the larger research network. Probiotics interaction, combined toxicity, and gut microbiota modification are noteworthy new themes that indicate quickly changing research boundaries in the field (Figure 8). Figure 8 shows thematic map based on author keywords showing conceptual structure of the field from Scopus dataset.

### 3.9 Keyword co-occurrence and emerging hotspots

**Figure 9:**
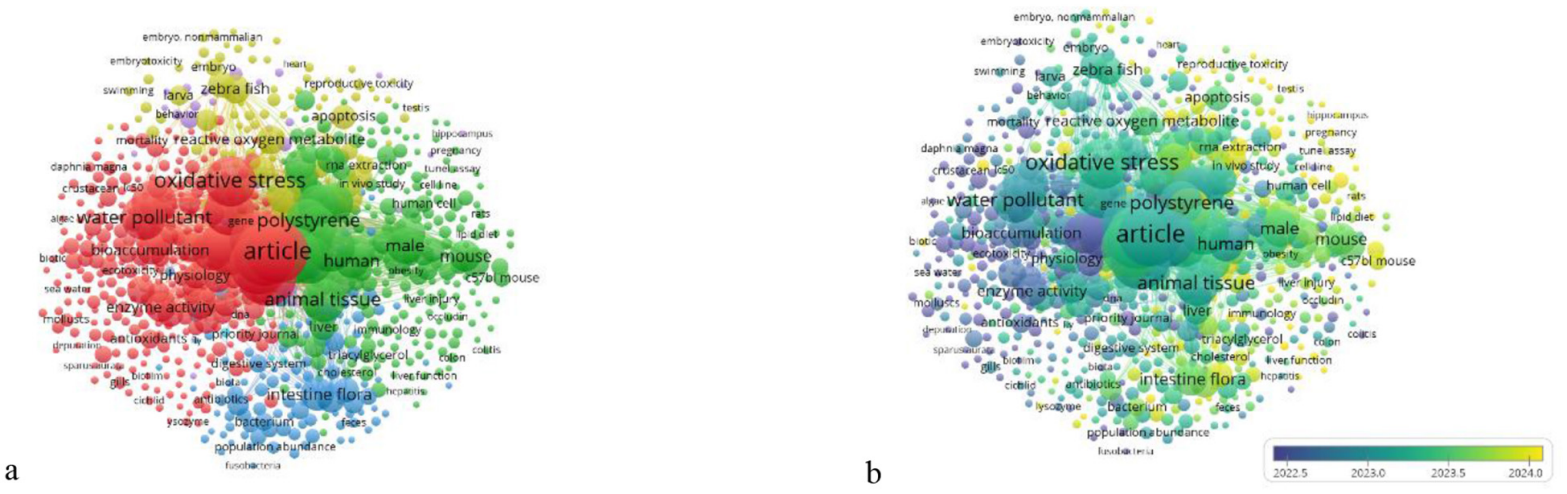
**(a)** Keyword co-occurrence network revealing four major research clusters: mammalian metabolic toxicity, gut microbiota dysbiosis, aquatic ecotoxicology, and emerging human health risk. Node size reflects keyword frequency and link thickness indicates co-occurrence strength. **(b)** Overlay visualization showing temporal evolution of research hotspots.

Four significant topic clusters were identified via keyword co-occurrence analysis. Mammalian metabolic toxicity research, especially liver dysfunction and high-fat diet interaction models, represented the largest cluster. A rising mechanistic focus was indicated by a second significant cluster that focused on host-microbe interactions and gut microbiota dysbiosis. The third cluster, which was defined by studies of aquatic organisms and toxicity evaluations based on enzymes, represented the field's ecotoxicological roots. The human health and cardiometabolic risk cluster was much smaller, with fewer studies focusing on human health and cardiometabolic outcomes. A recent trend toward lipid metabolism, gut-liver axis research, and omics-driven studies was further illustrated by overlay visualization (Figures 9a, b). The significant roles of oxidative stress, microplastics, nanoplastics, and gut microbiota interactions in the area were further confirmed by the keyword co-occurrence network constructed from the Web of Science dataset, which confirm the identified thematic structure.

### 3.10 Intellectual base and research front

**Figure 10:**
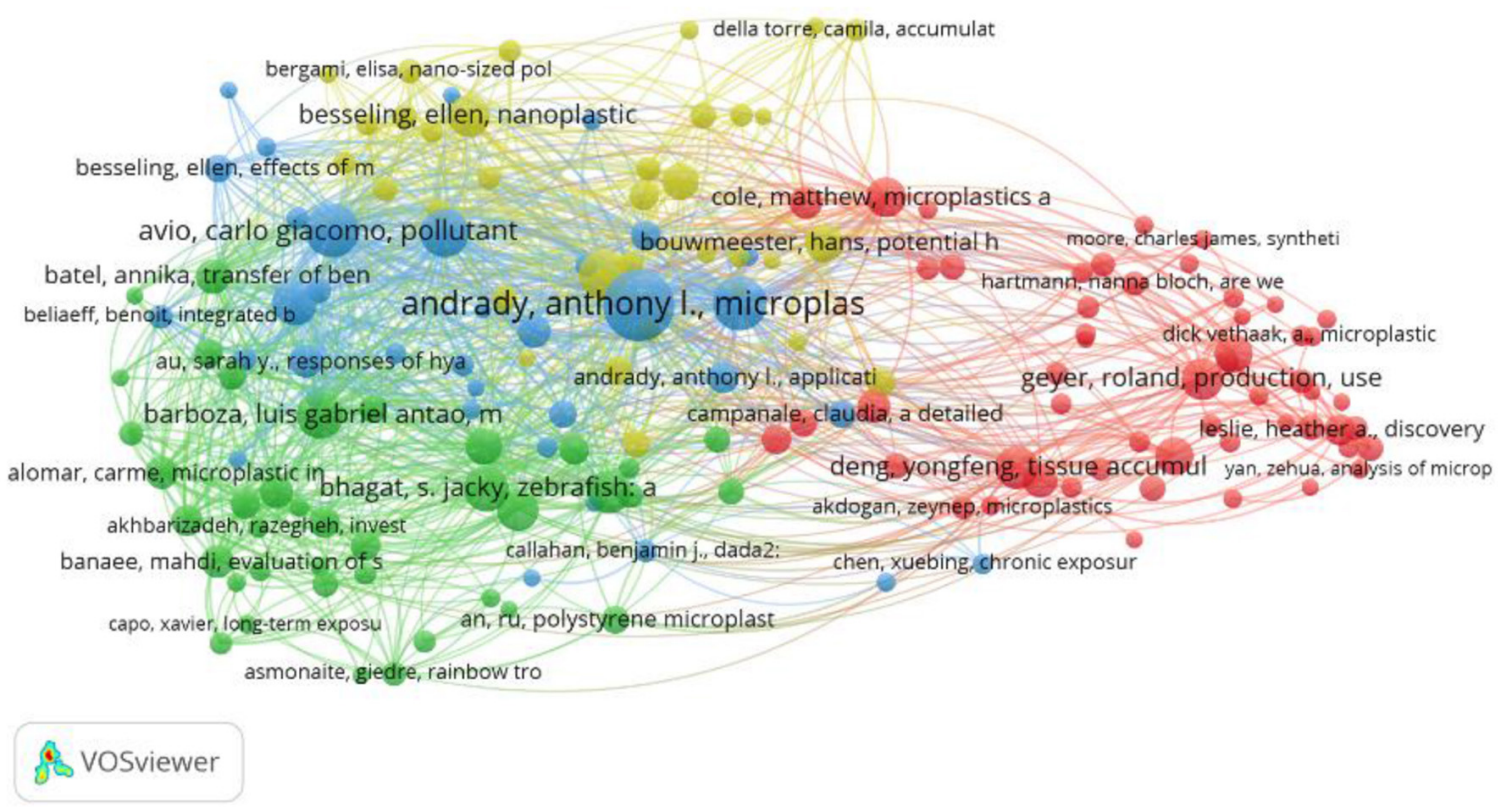
Co-citation network illustrating the intellectual base of the field.

Three main intellectual foundations were established through co-citation analysis: studies on early microplastic ingestion, studies on environmental dispersal and biodegradation, and evaluations of global plastic production and pollution. This suggests that current research on metabolic health has developed from foundation mostly focused on environmental contamination (Figure 10).

### 3.11 Bibliographic coupling

**Figure 11:**
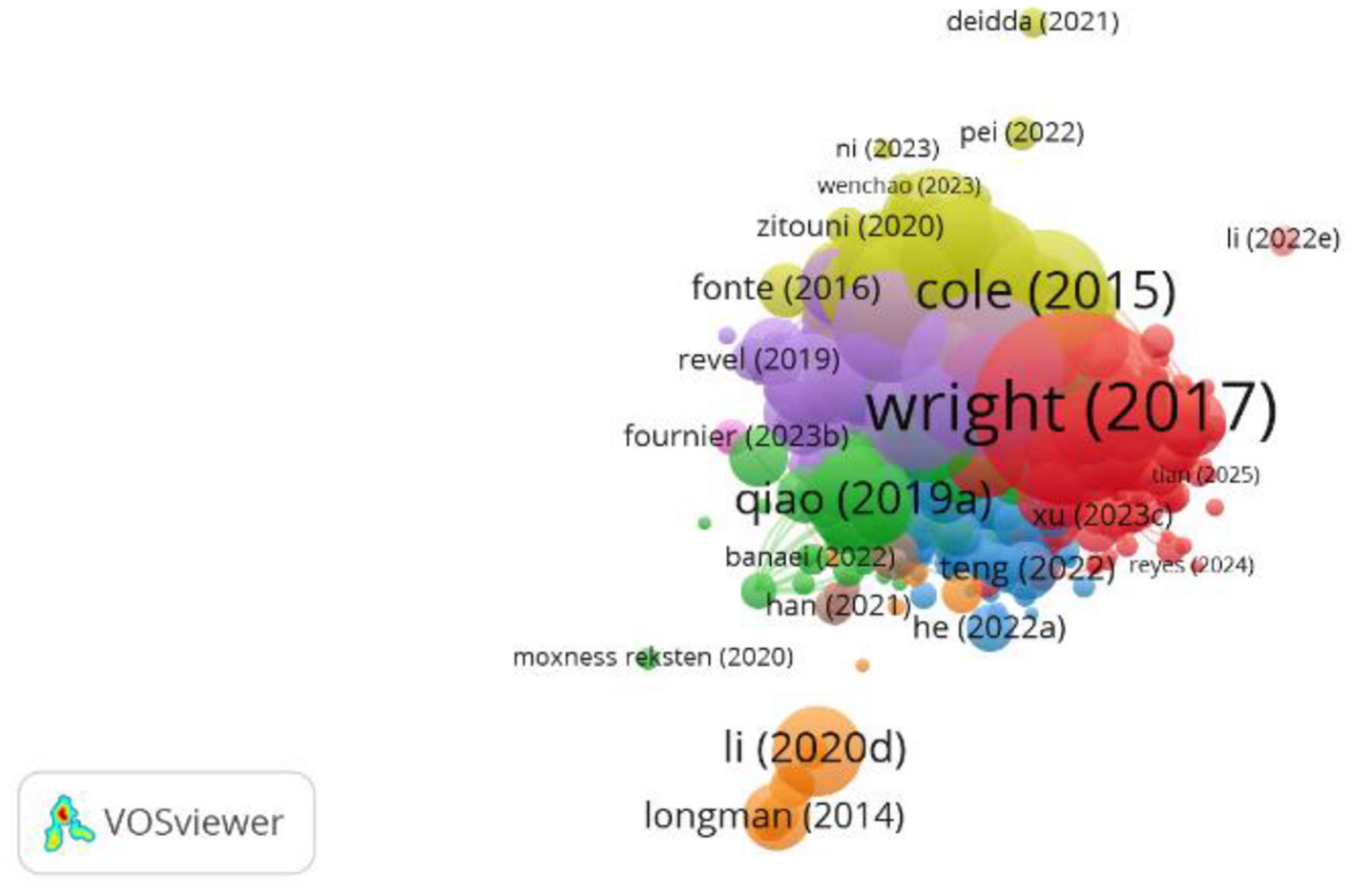
Bibliographic coupling network representing the current research front.

Current research is focused on three primary fronts, according to bibliographic coupling: gut microbiota disturbance, mammalian metabolic toxicity, and previous ecotoxicological studies. A notable a smaller representation of human epidemiological studies was observed is shown by the relative paucity of human epidemiology studies (Figure 11).

### 3.12 Biomarker domain evolution

A summary of biomarkers against biological systems and important mechanisms is shown in Table 3. Oxidative stress-related biomarkers represented the most frequently occurring domain, followed by lipid metabolism and inflammatory pathways, while glucose/insulin-related biomarkers were less frequently reported. An increased frequency of oxidative stress-related biomarkers was observed in studies involving nanoplastic exposure. Metabolic health-related outcomes were increasingly represented in studies associated with these biomarker domains. However, controlled animal research continues to dominate the present body of evidence, with comparatively little epidemiological validation and biomonitoring in humans. This translational gap emphasizes how urgently longitudinal cardiometabolic risk assessment and standardized human exposure biomarkers are needed. This analysis reflects thematic representation rather than quantitative biomarker effect size or biological validation.

**Table 3:** Presents illustrative examples of highly cited studies and does not represent a systematic biomarker extraction across the full dataset.

Top-cited paper (Title)	Year	Journal	Citations	Study type/model	Plastic type (from title)	Mechanistic focus	Biomarker domains indicated
Uptake and Accumulation of Polystyrene Microplastics in Zebrafish (Danio rerio) and Toxic Effects in Liver (16)	2016	Environmental Science & Technology	1846	Aquatic model	Polystyrene	Oxidative stress; Inflammation; Lipid metabolism	Oxidative stress; Inflammation; Lipids
Tissue accumulation of microplastics in mice and biomarker responses suggest widespread health risks of exposure (9)	2017	Scientific Reports	1362	Rodent	Microplastics	Oxidative stress; Lipid metabolism	Oxidative stress; Lipids
Polystyrene microplastics induce potential toxicity through the gut-mammary axis (17)	2018	Science of the Total Environment	918	Rodent	Polystyrene	Gut microbiome; Lipid metabolism; Glucose/insulin	Glucose/insulin; Lipids
Microplastics induce intestinal inflammation, oxidative stress, and disorders of metabolome and microbiome in zebrafish (18)	2019	Science of the Total Environment	837	Aquatic model	Microplastics	Gut microbiome; Oxidative stress; Inflammation; Barrier/endotoxemia	Oxidative stress; Inflammation; Lipids
Accumulation of different shapes of microplastics initiates intestinal injury and gut microbiota dysbiosis in the gut of zebrafish (11)	2019	Chemosphere	819	Aquatic model	Microplastics	Gut microbiome; Oxidative stress; Inflammation; Lipid metabolism	Oxidative stress; Inflammation; Lipids
Human health and ocean pollution (19)	2020	Annals of Global Health	470	Human/Population	Unspecified	Oxidative stress; Glucose/insulin; Barrier/endotoxemia	Not specified
Nanoplastics Cause Neurobehavioral Impairments, Reproductive and Oxidative Damages, and Biomarker Responses in Zebrafish: Throwing up Alarms of Wide Spread Health Risk of Exposure (20)	2020	Int. Journal of Molecular Sciences	382	Aquatic model	Nanoplastics	Oxidative stress; Lipid metabolism	Oxidative stress; Lipids
Microplastics induce transcriptional changes, immune response and behavioral alterations in adult zebrafish (21)	2019	Scientific Reports	352	Rodent	Microplastics	Lipid metabolism	Lipids
Maternal Polystyrene Microplastic Exposure during Gestation and Lactation Altered Metabolic Homeostasis in the Dams and Their F1 and F2 Offspring (22)	2019	Environmental Science & Technology	321	Rodent	Polystyrene	Gut microbiome; Lipid metabolism	Lipids; Barrier

## 4 Discussion

The inclusion of Web of Science data further strengthens the reliability of the bibliometric findings. Cross-database validation revealed highly consistent publication trends, research clusters, and thematic evolution patterns between Scopus and WoS datasets. This concordance suggests that the observed expansion of microplastic-metabolic health research is not an artifact of database coverage but reflects a genuine global shift toward mechanistic investigation of environmental contaminants and metabolic disease pathways.

This bibliometric analysis shows that over the past 10 years, research on the relationship between metabolic health and micro- and nanoplastics (MNPs) in the food chain has rapidly expanded and conceptualized. Growing interdisciplinary concern about the health effects of global plastic contamination is reflected in the substantial rise of publications after 2019. The area has gradually shifted toward mechanistic studies examining host physiological disturbance, whereas early research mostly concentrated on environmental occurrence and ecotoxicological implications (2, 3).

The field's conceptual foundation in pollution research is highlighted by the prevalence of environmental and toxicological publications among the most productive sources. Nonetheless, growing contributions from molecular and biomedical publications point to a move toward frameworks focused on health, which is in line with the growing understanding that plastic particles might be new environmental health hazards (14). This change is in line with the environmental persistence and worldwide manufacturing patterns of plastics, which have increased worries about long-term low-dose exposure (11).

The development of the microplastic–gut microbiota nexus as a key “motor theme” in the field's conceptual framework is among the study's most notable discoveries. Orally consumed MNPs interact with the intestinal epithelium, change microbial diversity, and compromise barrier integrity, according to an increasing number of experimental studies (8, 9). Systemic metabolic abnormalities, such as dyslipidaemia and glucose intolerance, frequently accompany these changes.

A common concept that connects environmental exposure to metabolic disorders is the gut-liver axis. Increased intestinal permeability, endotoxemia, and inflammatory signaling may be facilitated by changes in microbial composition, which could lead to insulin resistance and hepatic fat buildup. According to research on animals, exposure to microplastics can cause oxidative stress and deregulation of genes involved in lipid metabolism in hepatic tissue (10, 11). The field's conceptual development from descriptive exposure assessment to mechanistic systems biology is shown in the integration of microbiome and metabolic endpoints.

Nanoplastics, oxidative stress, and lipid metabolism were shown to be a very prominent but relatively underdeveloped subject in the thematic structure. Because of their nanoscale size, larger surface area, and higher potential for cellular absorption, nanoplastics may have improved biological reactivity. According to several studies, exposure to nanoplastics causes the production of reactive oxygen species (ROS), mitochondrial dysfunction, and changes in lipid metabolism (10).

Across experimental settings, oxidative stress seems to be a convergent process. Methodological heterogeneity is still a major drawback, though. Cross-study comparability and quantitative risk assessment are made more difficult by variations in particle size, polymer type, surface functionalization, and dose metrics. According to Koelmans et al. (15, 16), harmonized dosage measurements and standardized particle characterization are crucial for improving microplastic risk assessment. The application of mechanistic results in regulatory contexts is still limited in the absence of such harmonization.

Bibliographic coupling analysis shows that only a small amount of human epidemiological research has been conducted, despite growing mechanistic insights from rodent and aquatic models. Although human biological matrices, such as blood and lung tissue, have been found to include microplastics (6, 7), there is a dearth of solid longitudinal data connecting food exposure to cardiometabolic outcomes.

This translational gap is highlighted by the comparatively tiny and less connected human health cluster found in the keyword network. The majority of metabolic results come from carefully monitored laboratory exposures, frequently using amounts higher than those thought to be required by humans. Furthermore, estimates of food exposure are still erratic and unclear (4, 5). Causal inference is still restricted in the absence of standardized epidemiological frameworks and proven biomarkers for human exposure. It will take concerted efforts to standardize exposure measurement, create trustworthy biomonitoring techniques, and incorporate metabolic end points into population-based cohort studies in order to close this gap.

Oxidative stress markers, lipid metabolic endpoints, inflammatory cytokines, and glucose/insulin dysregulation are becoming more and more important, according to biomarker-domain mapping. This convergence highlights the increasing research focus on biological mechanisms potentially associated with metabolic disturbance. Following exposure to microplastics, animal studies consistently show increased levels of inflammatory mediators and oxidative stress indicators (8, 10).

Nonetheless, there is still a great deal of variation in the methods used for tissue sample, reporting, and biomarker selection. Reproducibility and cross-study comparability are restricted by inconsistent reporting of polymer identity, particle size distribution, and exposure time. The creation of a minimal reporting checklist that includes exposure measures, standardized particle characterization, and a core metabolic biomarker panel will greatly improve methodological comparability and transparency. Moving from mechanistic plausibility to evidence-based risk assessment frameworks requires this kind of harmonization.

Increased awareness that environmental pollutants may interact with non-communicable disease pathways is reflected in the growth of MNP–metabolic research. However, exposure quantification, toxico kinetics, and dose-response modeling must be integrated in order to convert experimental results into public health recommendations.

Microplastic risk assessment frameworks are still in the early phases of development (16). Accurate dietary exposure assessment is hampered by analytical constraints in detecting nanoscale particles inside complex food matrices. Furthermore, the majority of experimental research used high-dose or short-term exposure paradigms, which could not accurately represent low-level, chronic human exposure situations. To elucidate causative pathways and guide regulatory decision-making, future research should give priority to multi-tissue metabolic profiling, longitudinal human investigations, and environmentally realistic dosage models.

## 5 Limitations

There are various restrictions on this study. First, the bibliometric study might have overlooked pertinent regional literature because it was limited to English-language publications and the Scopus database and Wos database. Second, self-citation patterns and publication age have an impact on citation metrics. Third, biomarker-domain mapping indicates thematic emphasis rather than quantifiable biomarker prevalence because it was based on keyword mining of titles, abstracts, and author keywords rather than full-text extraction. Additionally, the exclusion of environmental matrices such as wastewater, soil, and sediment may introduce selection bias by underrepresenting indirect pathways through which MNPs enter the food chain. However, the convergence of results from bibliographic coupling, co-citation networks, theme mapping, and performance analysis offers a strong triangulation of the field's research trajectory and intellectual structure. The absence of full-text biomarker extraction and lack of quantitative synthesis limit the ability to infer biomarker prevalence or effect magnitude.

## 6 Conclusion

Research on micro- and nanoplastics in the food chain and metabolic health has entered a phase of rapid expansion and conceptual consolidation, as this bibliometric analysis shows. The knowledge framework is now firmly focused on the mechanistic pathways through which MNP exposure has been explored in relation to metabolic dysfunction, specifically through oxidative stress amplification, lipid metabolic dysregulation, and disturbance of the gut flora. A clear move away from investigations of merely environmental occurrences and toward integrative host-microbe and metabolic frameworks is confirmed by thematic evolution. A continuous global research direction is reflected in the Scopus dataset's rapid increase and thematic change, as confirmed by cross-database validation using Web of Science.

However, human epidemiological and clinical validation is relatively weak, and the evidence base is still significantly biased toward controlled animal and ecotoxicological models. The biomarker synthesis indicates significant variety in biomarker selection and reporting techniques, but it also emphasizes the increasing convergence around oxidative, lipid, and inflammatory endpoints. Translational risk assessment and cross-study comparability are restricted by these shortcomings.

## Data Availability

The raw data supporting the conclusions of this article will be made available by the authors, without undue reservation.

## References

[1] Geyer R Jambeck JR Law KL. Production, use, and fate of all plastics ever made. Science advances. (2017) 3:e1700782. doi: 10.1126/sciadv.1700782 28776036 PMC5517107

[2] Thompson RC Olsen Y Mitchell RP Davis A Rowland SJ John AW . Lost at sea: where is all the plastic? Science (New York, NY). (2004) 304:838. doi: 10.1126/science.1094559 15131299

[3] Cole M Lindeque P Halsband C Galloway TS. Microplastics as contaminants in the marine environment: a review. Mar Pollut Bull. (2011) 62:2588–97. doi: 10.1016/j.marpolbul.2011.09.025 22001295

[4] Cox KD Covernton GA Davies HL Dower JF Juanes F Dudas SE. Human consumption of microplastics. Environ Sci Technol. (2019) 53:7068–74. doi: 10.1021/acs.est.9b01517 31184127

[5] EFSA Panel on Contaminants in the Food Chain (CONTAM). Presence of microplastics and nanoplastics in food, with particular focus on seafood. EFSA journal. EFSA J. (2016) 14: e04501. doi: 10.2903/j.efsa.2016.4501 PMC11847996 40007823

[6] Amato-Lourenço LF Carvalho-Oliveira R Júnior GR Dos Santos Galvão L Ando RA Mauad T. Presence of airborne microplastics in human lung tissue. J Hazard Mater. (2021) 416:126124. doi: 10.1016/j.jhazmat.2021.126124 34492918

[7] Leslie HA van Velzen MJM Brandsma SH Vethaak AD Garcia-Vallejo JJ Lamoree MH. Discovery and quantification of plastic particle pollution in human blood. Environ Int. (2022) 163:107199. doi: 10.1016/j.envint.2022.107199 35367073

[8] Jin Y Lu L Tu W Luo T Fu Z. Impacts of polystyrene microplastic on the gut barrier, microbiota and metabolism of mice. Sci Total Environ. (2019) 649:308–17. doi: 10.1016/j.scitotenv.2018.08.353 30176444

[9] Lu L Wan Z Luo T Fu Z Jin Y. Polystyrene microplastics induce gut microbiota dysbiosis and hepatic lipid metabolism disorder in mice. Sci Total Environ. (2018) 631-632, 449–458. doi: 10.1016/j.scitotenv.2018.03.051 29529433

[10] Deng Y Zhang Y Lemos B Ren H. Tissue accumulation of microplastics in mice and biomarker responses suggest widespread health risks of exposure. Sci Rep. (2017) 7:46687. doi: 10.1038/srep46687 28436478 PMC5402289

[11] Qiao R Deng Y Zhang S Wolosker MB Zhu Q Ren H . Accumulation of different shapes of microplastics initiates intestinal injury and gut microbiota dysbiosis in the gut of zebrafish. Chemosphere. (2019) 236:124334. doi: 10.1016/j.chemosphere.2019.07.065 31310986

[12] Aria M Cuccurullo C. Bibliometrix: An R-tool for comprehensive science mapping analysis. J Informetr. (2017) 11:959–75. doi: 10.1016/j.joi.2017.08.007

[13] Donthu N Kumar S Mukherjee D Pandey N Lim WM. How to conduct a bibliometric analysis: An overview and guidelines. J Bus Res. (2021) 133:285–296. doi: 10.1016/j.jbusres.2021.04.070

[14] Wright SL Kelly FJ. Plastic and Human Health: A Micro Issue? Environ Sci Technol. (2017) 51:6634–47. doi: 10.1021/acs.est.7b00423 28531345

[15] Koelmans AA Redondo-Hasselerharm PE Nor NHM Vera NR Svenja MM Merel K. Risk assessment of microplastic particles. Nat Rev Mater. (2022) 7:138–52. doi: 10.1038/s41578-021-00411-y

[16] Lu Y Zhang Y Deng Y Jiang W Zhao Y Geng J . Uptake and Accumulation of Polystyrene Microplastics in Zebrafish (Danio rerio) and Toxic Effects in Liver. Environ Sci Technol. (2016) 50:4054–60. doi: 10.1021/acs.est.6b00183 26950772

[17] Wang Z Wang S Liu S Li F Bu Q An X. Polystyrene microplastics induce potential toxicity through the gut-mammary axis. npj Sci Food. (2025) 9:139. doi: 10.1038/s41538-025-00517-5 40659623 PMC12259839

[18] Qiao R Sheng C Lu Y Zhang Y Ren H Lemos B. Microplastics induce intestinal inflammation, oxidative stress, and disorders of metabolome and microbiome in zebrafish. Sci Total Environ. (2019) 662:246–53. doi: 10.1016/j.scitotenv.2019.01.245 30690359

[19] Landrigan PJ Stegeman JJ Fleming LE Allemand D Anderson DM Backer LC . Human Health and Ocean Pollution. Annals of global health. (2020) 86:151. doi: 10.5334/aogh.2831 33354517 PMC7731724

[20] Sarasamma S Audira G Siregar P Malhotra N Lai YH Liang ST . Nanoplastics cause neurobehavioral impairments, reproductive and oxidative damages, and biomarker responses in zebrafish: throwing up alarms of wide spread health risk of exposure. Int J Mol Sci. (2020) 21:1410. doi: 10.3390/ijms21041410 32093039 PMC7073134

[21] Limonta G Mancia A Benkhalqui A Bertolucci C Abelli L Fossi MC Panti C. Microplastics induce transcriptional changes, immune response and behavioral alterations in adult zebrafish. Sci Rep. (2019) 9:15775. doi: 10.1038/s41598-019-52292-5 31673028 PMC6823372

[22] Luo T Wang C Pan Z Jin C Fu Z Jin Y. Maternal polystyrene microplastic exposure during gestation and lactation altered metabolic homeostasis in the dams and their F1 and F2 Offspring. Environ Sci Technol. (2019) 53:10978–92. doi: 10.1021/acs.est.9b03191 31448906

